# Interaction of Some Non-Platinum Metal Anticancer Complexes With Nucleotides and DNA and The Two-Pole Complementary Principle (TPCP) Arising Therefrom

**DOI:** 10.1155/MBD.1998.41

**Published:** 1998

**Authors:** Pin Yang, Maolin Guo

**Affiliations:** Institute of Molecular Science Shanxi University Taiyuan 030006 China

## Abstract

The binding modes of some non-platinum metal anticancer complexes, Cp_2_TiCl_2_, Cp_2_ZrCl_2_, (CH_3_)_2_SnCl_2_, (C_2_H_5_)_2_SnCl_2_, (C_2_H_5_)_2_SnCl_2_(phen) (phen=Phenanthroline) and cis-Ru^II^Cl_2_(DMSO)_3_ (DMSO) (cis-RDT) with nucleotides and DNA in aqueous solution at physiological pH values were investigated by various modern techniques. 5'-dGMP with Cp_2_TiCl_2_ or cis-RDT forms chelate complexes in which both N_7_ and phosphate of dGMP bind to the metal center. Whereas Cp_2_ZrCl_2_ and all the diorganotin compounds can bind dGMP only via the phosphate group. The investigations of the interactions between Cp_2_TiCl_2_ or (C_2_H_5_)_2_SnCl_2_ and DNA indicate that there are two types of binding sites on DNA for Cp_2_TiCl_2_, i.e., the base nitrogen rings and the phosphate group, while (C_2_H_5_)_2_SnCl_2_ can bind to DNA only via the phosphate group. At last, by carefully comparing and analysing the binding modes-activity relationships of the above anticancer complexes and other non-platinum and platinum anticancer complexes, a hypothesis named “Two-Pole Complementary Principle” was put forward.


### INTERACTION OF SOME NON-PLATINUM METAL ANTICANCER COMPLEXES WITH NUCLEOTIDES AND DNA AND THE TWO-POLE COMPLEMENTARY PRINCIPLE (TPCP) ARISING THEREFROM

Pin Yang* and Maolin Guo

Institute of Molecular Science, Shanxi University, Taiyuan 030006, P. R. China

**ABSTRACT**

The binding modes of some non-platinum metal anticancer complexes, $Cp_2TiCl_2$, $Cp_2ZrCl_2$, $(CH_3)_2SnCl_2$, $(C_2H_5)_2SnCl_2$ , $(C_2H_5)_2SnCl_2$(phen) (phen=Phenanthroline) and cis-$Ru^{II}Cl_2(DMSO)_3$ (DMSO) (cis-RDT) with nucleotides and DNA in aqueous solution at physiological pH values were investigated by various modern techniques. 5'-dGMP with $Cp_2TiCl_2$ or cis-RDT forms chelate complexes in which both $N_7$ and phosphate of dGMP bind to the metal center. Whereas $Cp_2ZrCl_2$ and all the diorganotin compounds can bind dGMP only via the phosphate group. The investigations of the interactions between $Cp_2TiCl_2$ or $(C_2H_5)_2SnCl_2$ and DNA indicate that there are two types of binding sites on DNA for $Cp_2TiCl_2$ , i.e., the base nitrogen rings and the phosphate group, while $(C_2H_5)_2SnCl_2$ can bind to DNA only via the phosphate group. At last, by carefully comparing and analysing the binding modes-activity relationships of the above anticancer complexes and other non-platinum and platinum anticancer complexes, a hypothesis named "Two-Pole Complementary Principle" was put forward.

### 1. Introduction

Metal complexes are promising compounds for the design of new anticancer agents. Cis-diaminodichloro-platinum(II) (cisplatin) and cis-diamine (cyclobutane-1,1-dicarboxylato) platinum(II) (carboplatin) are the first antitumor drugs in clinical use[1]. In order to overcome clinical problems associated with the relatively limited activity of cisplatin and carboplatin against the broad spectrum of human malignancies, acquired resistance, and side effects, a lot of new non-platinum metal-based anticancer complexes have been developed. Among them, some compounds containing Ti、V、Mo、Ru as metal center atoms have shown good antitumor activity and remarkable low toxicity; and some diorganotin(IV) complexes have been found to be effective against lymphocytic leukemia P388 cells in mice, which makes them interesting for possible clinical use[2-4].

Biological studies have shown that DNA is the primary cellular target for these metal-based drugs[3,5-8]. Intensive efforts of various research groups in recent years have contributed significantly toward an improved understanding of the mechanism of the antitumor activity of platinum complexes[1,9,10]. However, significantly less is known about the antitumor mechanisms of non-platinum metal-based complexes.

A main goal of our research has been to elucidate the aqueous coordination chemistry of non-platinum metal-based anticancer complexes with nucleotides and their DNA binding modes. These results should be valuable in understanding the mechanism of antitumor activity, as well as laying the foundation for rational design of more active and less toxic metal drugs.

In this paper, we present the reactions of some typical antitumor agents, $Cp_2TiCl_2$ (Cp= $\eta^5$-$C_5H_5$), $Cp_2ZrCl_2$, cis-$Ru^{II}Cl_2(DMSO)_3$ (DMSO) (cis-RDT), $(CH_3)_2SnCl_2$, $(C_2H_5)_2SnCl_2$ and $(C_2H_5)_2SnCl_2$(phen) (phen=Phenanthroline), with DNA and its components, and report their coordination chemistry investigations in aqueous solution with nucleotides, as well as their bindings to DNA under near physiological condition. The possible molecular mechanism modes of action are investigated and discussed as well as some rules of binding modes-activity relationships of metal antitumor drugs are inferred and discussed .

### 2. Experimental

The antitumor agents $Cp_2TiCl_2$(TDC), cis-RDT, $(CH_3)_2SnCl_2$, $(C_2H_5)_2SnCl_2$ and $(C_2H_5)_2SnCl_2$(phen) were prepared and purified according to the literature methods[11-14], $Cp_2ZrCl_2$ and $(CH_3)_2SnCl_2$, were purchased from Aldrich Chemical Company. The NMR recognition probe, [*trans*-$en_2Os(\eta^2$-$H_2)](CF_3SO_3)_2$ ,was a generous gift from Professor H. Taube and Dr. Zaiwei Li, Stanford University, CA, the nucleotides, 2'-deoxyguanosine-5'-monophosphat (dGMP), adenosine-5'-monophosphate (AMP) were purchased from Sigma Chemical Company. Ethidium bromide(EthBr) was obtained from Fluca(Buchs, Switzerland). All other reagents and solvents were analytical reagents grades.

$^1H$,$^{31}P$ NMR spectra were recorded on a Bruker AM-500MHz NMR spectrometer or a VXR-300 MHz NMR spectrometer in $D_2O$, proton chemical shifts are referenced to DSS as internal standard, and phosphorous chemical shifts are referenced to 85% $H_3PO_4$ as external standard. UV and CD spectra were recorded on a Shimadzu UV-365 spectrophotometer and a JASCO J-500C spectrophotometer using 1cm quartz cuvette. The fluorescence spectra were performed on a Hitachi Model-850 spectra fluorometer using 1 cm quartz cuvette , excitation wave length was set at 520 nm. The entrance and exit monochromator slit widths were adjusted to 0.5 nm . All pH measurements were carried out on a Beckman $\phi$ 50 pH meter. All reported pD values in $D_2O$ are corrected to pH readings(pD=pH + 0.44). The cyclic Voltammetric measurements were performed with a BAS-100A electrochemistry analytical instrument, an Ag/AgCl electrode was used as the reference electrode with Pt as the auxiliary electrode, and a hanging mercury drop electrode was chose as the working electrode.

### 3. Results and Discussion

The objective of the investigation was the solution characterization of aqueous antitumor metal agents-nucleotide coordination chemistry and the elucidating of the possible molecular mechanism mode of action between metal agents with DNA. We begin with a discussion of metal drugs aqueous coordination chemistry with dGMP using [*trans*-$en_2Os(\eta$-$H_2)](CF_3SO_3)_2$ as a $^1H$ NMR recognition probe followed by high-resolution $^1H$, $^{31}P$, $^{13}C$ NMR spectroscopy , and then we discuss the interactions between metal drugs with DNA under near physiological conditions. At last, the relationships between the modes of action of drugs and their anticancer activities and some rules are put forward.

### 3.1 *Aqueous metal agents coordination with dGMP*

The probe, [ *trans*-$en_2$ Os ( $\eta^2$-$H_2$) $]^{2+}$ (scheme1), as previously demonstrated[15], bind readily to a variety of biomolecules such as nucleotides with ligand, L, substituted by these biomolecules, that results in the direct coordination of $Os^{II}$ to the donor atoms(such as N,O) of the biomolecules. In each case the binding leads to characteristic $^1H$ NMR spectrum for the dihydrogen unit that appears in a spectral window in the range $\delta$ =0 to -20.

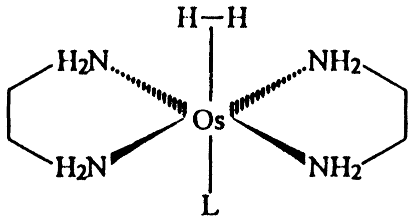

scheme1

When dGMP was added to solution of the probe in $D_2O$ at $25^0C$ each solute at 0.01 mol/ L, the phosphate oxygen and $N_7$ of dGMP both coordinated to the probe, the former being dynamically preferred ($K=3\times10^2$) and the interaction was complete after 10 min, while the latter is thermodynamically preferred ($K=2.9\times10^3$) and binding was complete after 24h. Figure 1

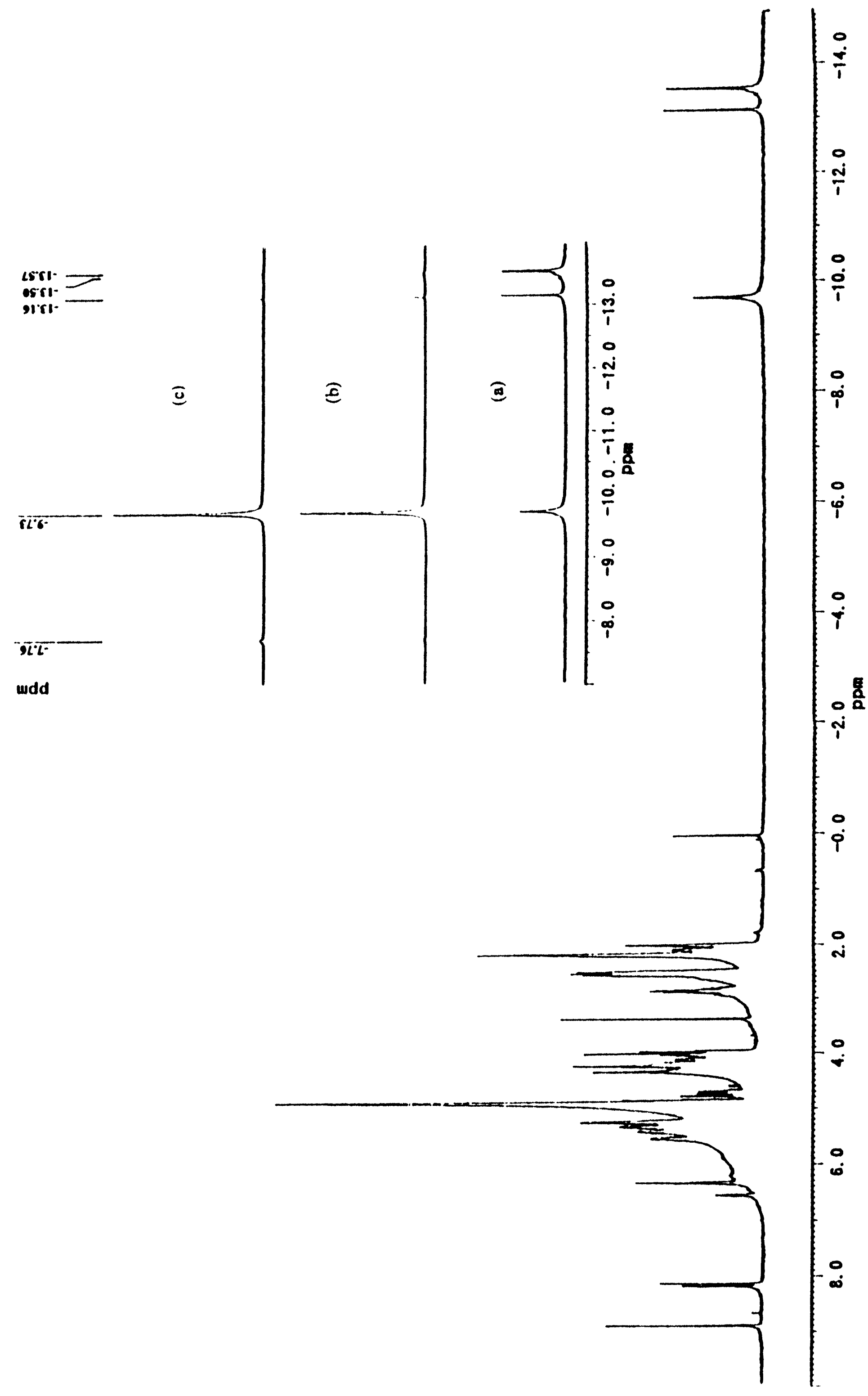

Figure 1. Proton NMR spectra (500MHz) of the probe with dGMP in $D_2O$ (pD=7.20, both 0.010mol/L)

(a) After 20 min (b) After 24 hours clearly shows the reaction process. The peaks at $\delta$=-13.16 and -13.56 grow in 10 min, corresponding to $D_2O$ binding and phosphate oxygen binding, respectively. Meanwhile, a peak at $\delta$ = -9.73, already discernible after 10min(Fig. 1a), continues to grows at the expense of the others. This peak is assigned to the $N_7$ binding. There is a competing reaction between $D_2O$ bonding, phosphate binding and $N_7$ binding, which is not completely at equilibrium in 10 min. The affinity of $Os^{II}$ for the $D_2O$ and phosphate is not high and the conversion from $D_2O$ binding and phosphate binding to $N_7$ binding is almost complete after 24h (Fig 1b). Usually the binding of an antitumor metallic agent to dGMP is via $N_7$ or the phosphate group of dGMP, therefore, if an antitumor metal agent is added to the probe-dGMP binary system, it will compete for the binding sites of dGMP with the probe, and the characteristic peaks due to binding of the probe with phosphate or $N_7$ of dGMP will change. According to these changes, the binding sites of the anticancer metal drugs to dGMP can be determined.

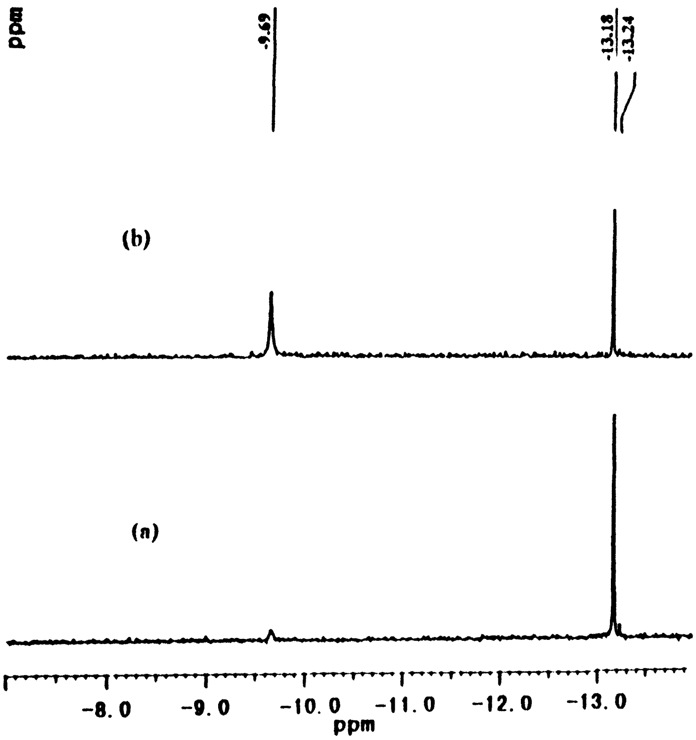

Figure 2. Proton NMR spectra (500MHz) of the probe with dGMP and $Cp_2TiCl_2$ in $D_2O$
(a) After 20 min (b) After 24 hours drug: dGMP: probe=1: 1: 1, dGMP 0.010mol/L, pD=6.14

When dGMP, $Cp_2TiCl_2$ and the probe were mixed at the same time at 25°C (Figure 2a、b), the peaks due to the phosphate binding(-13.56) disappears completely 10 min after mixing, and the peak due to $N_7$ binding is much lower than that of the probe-dGMP binary system. This indicates that Ti completely occupies phosphate oxygen sites and partially occupies $N_7$ sites of dGMP. After 24h, cis-isomer of probe binding to $N_7$ does not appear, which shows that Ti hinders the binding of probe to $N_7$. These indicate bindings of Ti to both phosphate and $N_7$.

While in the ternary system of $Cp_2ZrCl_2$-probe-dGMP, only the peaks due to the phosphate binding disappear, the peaks due to $N_7$ binding is not affected at all during the reaction process, suggesting Zr only coordination to phosphate of dGMP but not $N_7$.

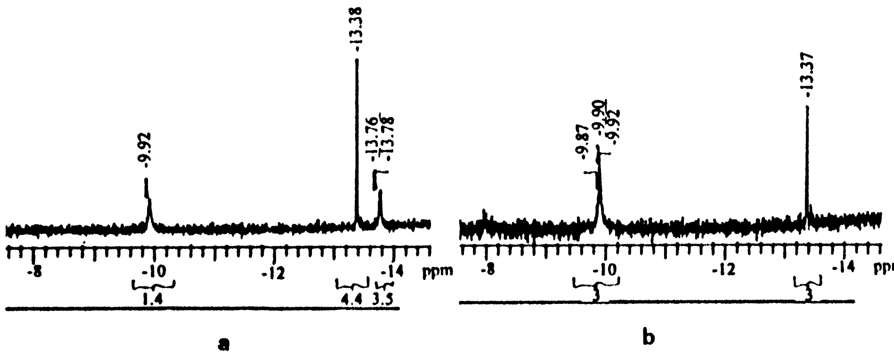

Figure 3. Proton NMR spectra (300MHz) of the probe with dGMP and *cis*-RDT in $D_2O$ (a) After 20 min (b) After 24 hours

In the ternary system of cis-Ru $(DMSO)_4Cl_2$-probe- GMP (Fig.3a、b) both of the peaks due to the phosphate binding and $N_7$ binding diminished obviously in intensity after 10 min (Fig.3a) and 24h compared with those of the GMP-probe binary system. This indicate that both phosphate oxygen and $N_7$ of dGMP are coordinated to $Ru^{II}$.

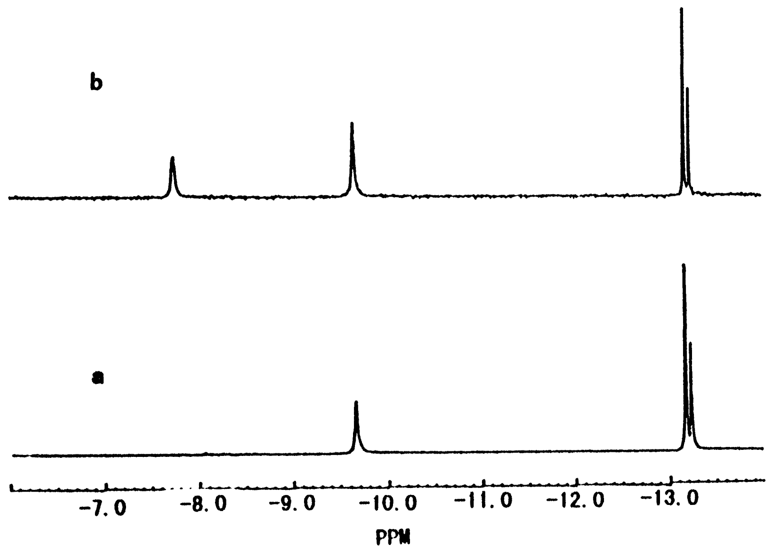

Figure 4. Proton NMR spectra (500MHz) of the probe with dGMP and $Et_2SnCl_2$ in $D_2O$
(a) After 20 min (b) After 24 hours drug: dGMP: probe=1: 1: 1, dGMP 0.005 mol/L, pD=7.44

As diorganotin agents, both fresh prepared and old prepared (has been prepared for three months) solutions of $Et_2SnCl_2$ were used. In the ternary system of $Et_2SnCl_2$(fresh)-probe-dGMP (Fig.4a、b), the case is just the same as that in $Cp_2ZrCl_2$-probe-dGMP trinary system.

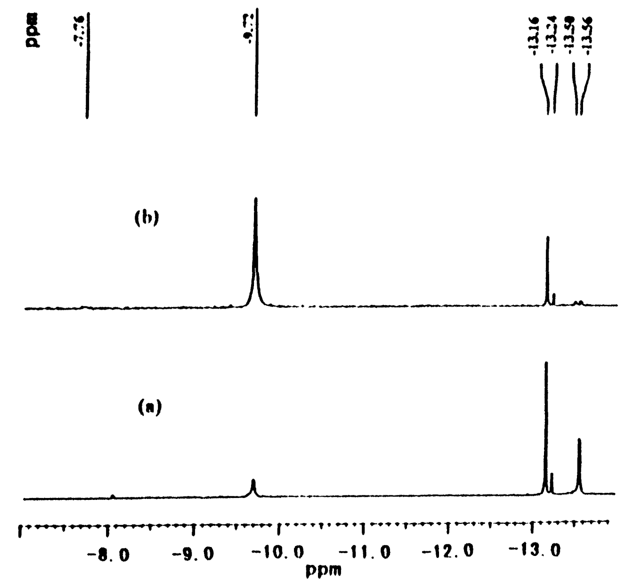

Figure 5. Proton NMR spectra (500MHz) of the probe with dGMP and $Et_2SnCl_2$ (have been prepared for long time) in $D_2O$ (a) After 20 min, (b) After 4 hours drug: dGMP: probe=1: 1: 1, dGMP 0.005 mol/L, pD=7.44

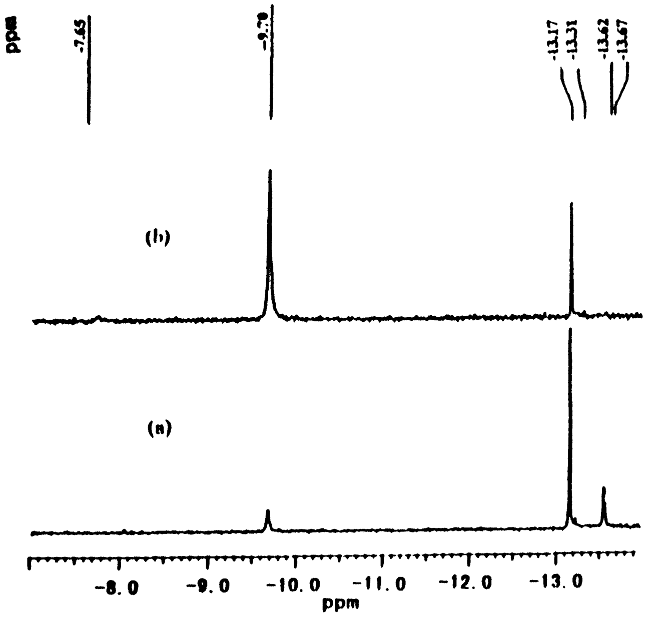

Figure 6. Proton NMR spectra (500MHz) of the probe with dGMP and $Et_2SnCl_2$(phen) in $D_2O$

(a) After 20 min (b) After 24 hours drug: dGMP: probe=1: 1: 1, dGMP 0.002mol/L, pD=6.44

When old $Et_2SnCl_2$ is used in place of fresh $Et_2SnCl_2$(Fig.5a、b), we find that old $Et_2SnCl_2$ does little effect on the binding of probe to phosphate or $N_7$. This suggest that fresh Sn can only coordinate to phosphate but not to $N_7$ of dGMP, while old Sn can coordinate to neither phosphate nor $N_7$ of d GMP. When fresh $Et_2SnCl_2$(phen) were in place of fresh $Et_2SnCl_2$, NMR results are shown in Fig.6a、b. We can find that $Et_2$ (phen)$Sn_2$(IV) can only bind to phosphate of dGMP but not to $N_7$.

In all the above figures, we can find a new peak at $\delta$ = -13.23 ppm appears after drug-dGMP interaction. The peak is assigned to $Cl^-$ binding to the probe. This indicates that all the above metal complexes dissociate in water with the cis-chloro atom as leaving group.

$^1H$, $^{31}P$, $^{13}C$ NMR data (Table 1) provide further support for the above results. $^1H$ NMR spectra of dGMP $Na_2$ alone shows a singlet at 7.93 ppm corresponding to the C(8) protons. When anticancer metal complex (0.01mol /L ) was incubated with 5'-dGMP (or 5'-GMP) in a 1：1 molar ratio in $D_2O$ at 25$^0$C, examination of reaction mixtures by $^1H$ NMR find that downfield position of the new $H_8$ signals appears for $Cp_2TiCl_2$, cis-RDT, but not for $Cp_2ZrCl_2$, $Et_2SnCl_2$, $Et_2SnCl_2$(phen). The down field shift of $H_8$ signals suggests $N_7$ coordination in $Cp_2TiCl_2$-dGMP system and cis-RDT-GMP system. $^{13}C$ NMR data indicate that the signals of C(6) and C(8) are not changed when dGMP have been incubated with $Et_2SnCl_2$ or $Et_2SnCl_2$(phen) for 24h, further demonstrating $N_7$ or $O_6$ of dGMP is not coordinated to Sn(IV).

Table 1. NMR spectroscopic data (ppm) for dGMP($Na_2$)(or GMP $Na_2$) involved in the reaction with metal anticancer complexes

| Compounds | H(8) | H(1') | $^{31}P$ | $^{13}C(8)$ | $^{13}C(6)$ |
|---|---|---|---|---|---|
| dGMPNa$_2$ | 7.93(s) | 6.08(d) | 3.81(s) | 115.33 | 158.15 |
| dGMPNa$_2$: $Et_2SnCl_2$ (1:1) | 7.87(s) | 6.10(d) | -1.09(s) | 115.58 | 158.44 |
| dGMPNa$_2$: $Et_2SnCl_2$(phen) (1:1) | 7.84(s) | 6.00(d) | 1.62(s) | — | — |
| dGMPNa$_2$: $Cp_2TiCl_2$ (1:1) | 8.43<br>8.14 | | 6.15 | | |
| dGMPNa$_2$ : $Cp_2ZrCl_2$ (1:1) | 7.98 | | 2.96<br>1.50 | | |
| dGMPNa$_2$ : cis-RDT (1:1) | 8.20<br>8.00 | | 12.9<br>11.29 | | |

s=singlet, d=doublet.

The $^{31}P$ NMR data also establish phosphate group coordination to all the metal drugs tested. The free dGMPNa$_2$ shows a singlet at 3.81ppm, in the $^{31}P$ NMR spectra, this peak is shifted either upfield or downfield upon addition of the five metal complexes, respectively, this suggest that the five metal complexes are directly bind to dGMP(or GMP) via phosphate group.

The above results suggest that the interactions between dGMP(GMP) with $Cp_2TiCl_2$ or cis-

RDT yield chelate complexes with $N_7$ and phosphate coordination, while interactions between dGMP with $Cp_2ZrCl_2$, $Et_2SnCl_2$, $Et_2SnCl_2$(phen) yield complexes with only phosphate coordination.

### 3.2 *Interactions between Metal Complexes and DNA*

By means of UV, CD, fluorescence spectra and CV, we investigated the interaction of two typical metal complexes, $Cp_2TiCl_2$ and $Et_2SnCl_2$, with calf thymus DNA and salmon DNA. The results show that there are two types of binding sites between DNA and $Cp_2TiCl_2$, one is the phosphate group of DNA and the other is the base nitrogen rings of DNA whereas only the phosphate group on DNA can binds to $Et_2SnCl_2$.

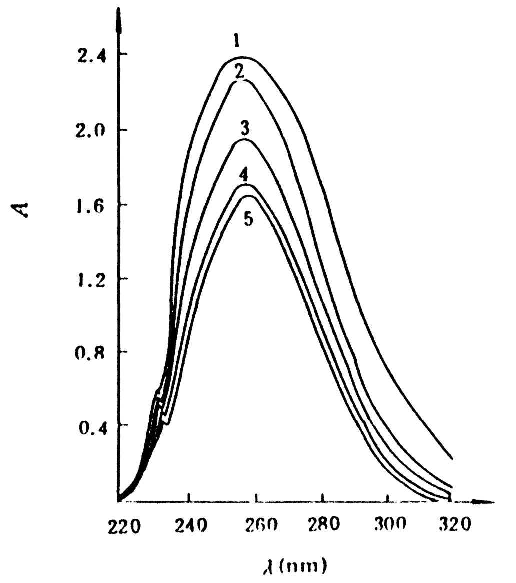

Figure 7. The effects of $Cp_2TiCl_2$ on the absorption spectra of calf thymus DNA, 1—5, $R_t$: 1.66, 0.83, 0.42, 0, 0.21

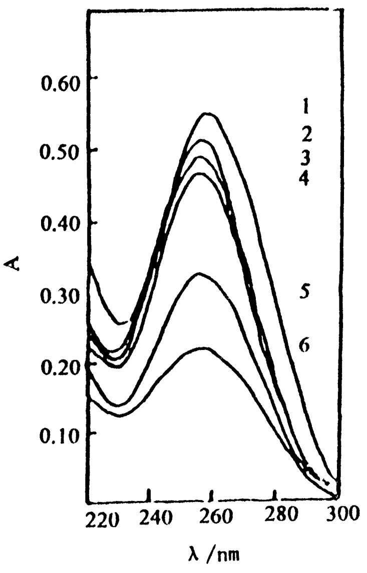

Figure 8. UV absorption change of the mixture of $Et_2SnCl_2$ and DNA with the drug concentrations. 1-6, $R_t$: 0, 0.2, 0.5, 1.0, 2.0, 4.0, respectively.

#### *UV spectra*

Figure 7 shows the absorption spectra of DNA alone in the presence of $Cp_2TiCl_2$ with various concentration in 0.1 mol/L NaCl(pH=7.2) after they reacted in dark at $37^0C$ for 48h (the absorption of $Cp_2TiCl_2$ solution as background ) . When $R_t$ ($=C_{TDC}/C_{DNA(P)}$) is low , typical "hypochromic effect " occurs, when $R_t$ is high, typical "hyperchromic effect" of DNA appears. Hypochromism results from the contraction of DNA in the helix axis, as well as from the change in conformation on DNA, while hyperchromism results from the damage of DNA double-helix structure. It is indicated from Fig.7 that $Cp_2TiCl_2$ binds to phosphate group of DNA backbone at low $R_t$ and to the base nitrogen rings of DNA at high $R_t$ to damage the duplex structure of DNA.

If $Cp_2TiCl_2$ is replace by $Et_2SnCl_2$, the UV spectra shown in Fig.8 demonstrates that $Et_2SnCl_2$ can only cause DNA hypochromism but not hyperchromism. This result indicates that $Et_2SnCl_2$ can induce the contraction in DNA helix but can not destroy the double helix structure of DNA. This result also reflect that $Et_2SnCl_2$ only binds with the phosphate group of DNA. In addition, we discover that the phosphate group ions can markedly inhibit the interaction of $Et_2SnCl_2$ with DNA. This also supports the conclusion that $Et_2SnCl_2$ only binds with the phosphate group of DNA.

### *CD spectra*

Fig.9 shows the CD spectra of DNA in the presence of $Cp_2TiCl_2$ with various concentrations in 0.01 mol/L $KNO_3$ after they reacted in the dark at $37^0C$ for 48h. The CD bands show no great change at low $R_t$(=0.25 or 0.50), but a small diminution of the intensity of negative bands as well as a little increase and blue shift of the positive bands. The changes suggest the transition of B-DNA to A-DNA[16], which indicates that DNA is contracted by $Cp_2TiCl_2$, supporting the conclusion of UV spectra.

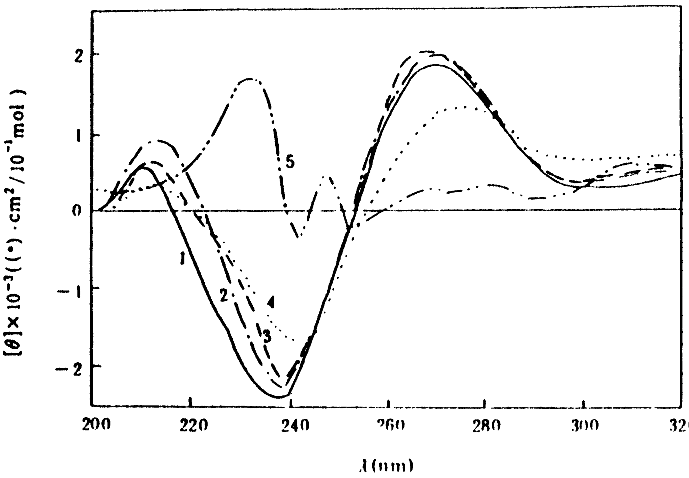

Figure 9. The effects of $Cp_2TiCl_2$ on the CD spectra of thymus DNA.
1—5, $R_t$ : 0, 0.25, 0.50, 1.0, 2.0.

The CD band changes inversely when $R_t$=1, and there is a red shift from 273nm to 279nm for positive CD band and 237nm to 243 nm for negative CD band, both losing intensity seriously, which suggests a decrease in helixity and denaturation of DNA [17,18], indicating that binding of $Cp_2TiCl_2$ with DNA base nitrogen rings occurs , when $R_t$=2, there is a little precipitate in the solution. The CD spectrum of the filtrate is recorded. The CD band changes greatly, the negative band almost disappears completely and a new positive band at 234 nm appears, which confirms that $Cp_2TiCl_2$ binds with the bases to cause the denaturation of DNA at high $R_t$.

***Fluorescence studies***

The measurement of EthBr binding to DNA metal complexes was studied by an increase of EthBr fluorescence [19]. The method essentially consists of titrating a given amount of DNA-metal complex with increasing concentration of EthBr and measuring the change in fluorescence intensity. The characteristics of the binding of EthBr to DNA can be expressed by Scatchard equation [19]:

$$r/C=K(n-r)$$

Here, r is the molar ratio of bound EthBr per nucleic acid phosphate, n the number of binding sites per nucleic acid, K, the intrinsic association constant to a site, and C, the free EthBr concentration. We can obtain straightline Scatchard plots using a function of r, and distinguish the drug action mode by using the Scatchard plots in the presence of drug [19,20].

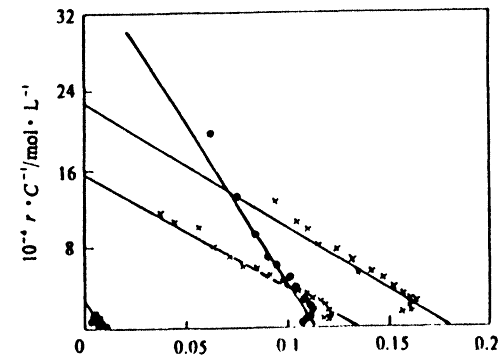

Figure 10. Scatchard plots. With the decrease of intercept on r axis of the curves, $R_t$: 0, 0.25, 0.5, 1.0 and 2.0, respectively.

Fluorescence Scatchard plots for the binding of EthBr to solution DNA in the presence of varying concentration of $Cp_2TiCl_2$ are given in Fig.10. At low concentration of drug ($R_t$=0.25), we observed uncompetitive inhibition of EthBr binding to DNA [20]. Here, only the intercept of the abscissa, which is the number of binding sites per nucleotide (n), decreases, whereas the slope or the association constant (K) remains constant. This uncompetitive inhibition is probably due to binding of the drug to the phosphate group of DNA. At high concentration ($R_t$=0.5, 1), the Scatchard plots fit mixed mode in which K and n values both change, this means both competing and uncompeting modes exist at these $R_t$s. This indicates that $Cp_2TiCl_2$ has two types of binding sites on DNA; that is say , besides binding to the phosphate, $Cp_2TiCl_2$ may bind to DNA on it's base nitrogen rings . Especially at $R_t$=0.5, the Scatchard plots are similar to that of RNA-EthBr or A-DNA-EthBr complexes[19]. This further implies that the double helix of DNA decreases, the secondary structure has changed with the conformation transferring from B-form to A-form. This conclusion is also confirmed by CD spectra shown above. At $R_t$=1, there are few binding sites(n < 0.01) on DNA for EthBr, indicating the double helix structure of DNA has been badly damaged .

***CV study***

CV method is one of the powerful methods that elucidate the interaction modes between drugs and DNA[21]. Native DNA is not reducible at the mercury electrode because the stability of the intact double helix makes the reducible bases inaccessible to the electrode. In Hepes buffer, pH=7.0 , the addition of DNA cause the peak potentials of $Et_2SnCl_2$ , $E_{pc}$ and $E_{pa}$ both to be shifted to more positive values, while the peak currents keep the same, as shown in Figure 11. The obvious positive shift of peak potentials indicates the binding between drug and the phosphate of DNA[21,22]. Here, this mode may be the binding between Sn and DNA via only the phosphate group of DNA .

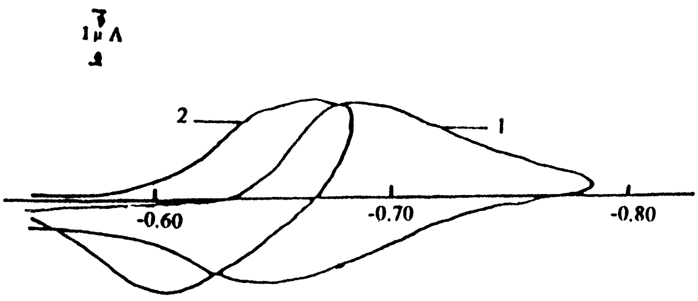

Figure 11. Cyclic voltammogram of 0.45 mM $Et_2SnCl_2$
(1) in the absence and (2)in the presence of DNA (0.45 mM nucleotide phosphate).
Supporting electrolyte 50 mM $KNO_3$, 5 mM Hepes, pH=7.0, Sweep rate, 200 mv/s.

Making a comparison between the binding modes with dGMP and DNA for the five antitumor metal complexes, we can find that $Cp_2TiCl_2$ and cis-RDT are similar, they both bind to dGMP(or DNA) by the metal center coordinating with the phosphate oxygen and $N_7$ of dGMP, and the binding modes of $Cp_2ZrCl_2$, $(C_2H_5)_2SnCl_2$ and $(C_2H_5)_2SnCl_2$(phen) are similar, they all only bind to dGMP (or DNA) by the metal center coordinating with the phosphate oxygen . It is very interesting if we relate the binding modes with their anticancer activities. $Cp_2TiCl_2$ and cis-

RDT both are highly active anticancer agents with a wide antitumor spectrum, while $Cp_2ZrCl_2$, $(C_2H_5)_2SnCl_2$ and $(C_2H_5)_2SnCl_2$(phen) are not good anticancer agents, $(C_2H_5)_2SnCl_2$ and $(C_2H_5)_2SnCl_2$(phen)can only inhibit P388 cell growth and $Cp_2ZrCl_2$ has no activity at all. Studies on the binding modes of other high active anticancer metal agents, such as cisplatin and $Cp_2MoCl_2$, also revealed that they can both bind with phosphate group and base $N_7$ of DNA or nucleotides[23,24]. Although binding of metal ions to the sugar ring oxygen atoms has rarely been observed, Pt-phosphate interactions do occur in certain cases[25-27]. The role of phosphate under physiological conditions seems to be only secondary for coordination; nevertheless, it is quite important for hydrogen bonding, and the binding between metal ion and phosphate might be the first step of metal agents-DNA(or nucleotides) interactions, just as in the case of $Cp_2TiCl_2$-DNA interaction. Definitive NMR evidence was presented that there has $N_7, \alpha$ $PO_4$ chelate species[27] in the mixture of 5'-GMP and cisplatin. Marzilli and coworkers[28] reported conclusive evidence from a multinuclear NMR study, supported by a molecular mechanics calculation, that in dilute (5-30 mM) neutral $D_2O$ solutions, the preferred 1:1 complexes formed between cis-$Pt(ND_2CH_3)_2(D_2O)$ and purine 5'-NTP, are monomeric macrochelates of the type cis-Pt( $ND_2CH_3)_2$ (5'-NTP-$N_7$, $\gamma$ PO) where the nucleotide is bound via $N_7$ and an oxygen atom of the $\gamma$ -phosphate group. Such species were observed as intermediates during the course of the reaction when r(Pt/NTP)= 0.5 and were the major products formed at $2 \geq r \geq 1$. Indeed, more and more unambiguous $N_7$, $\alpha$ PO chelate complexes have been reported by studying interactions between active anticancer metal complex and nucleotides. Marks and coworkers [24,29] has reported the interactions between anticancer $Cp_2MoCl_2$ and 5'-AMP yield a $N_7$ , $\alpha$ PO chelate $Mo(C_5H_5)_2$(5'-AMP-$N_7$,$\alpha$ PO). The crystal structure of $[Cp_2Mo(5'\text{-dGMP})]_2$ provides a further example of binuclear $N_7$ ,PO chelation[24]. Interactions between antimetastatic agents trans-$RuCl_2$ $(DMSO)_4$ and 5'-dGMP also forms $N_7$, $\alpha$ $PO_4$ chelation[30]. So that the binding modes with DNA of a metal complex seem play a key role in its anticancer activity. It seems all highly active anticancer metal drugs has the ability to bind with both phosphate group and nitrogen sites on bases of DNA. As everybody knows, chemical activities of a compound are determined by its molecular structure. So we should make further analysis on the relationships between the structure of metal anticancer agents, the binding modes with DNA and their anticancer activities.

DNAs are the target molecules for most of the metal anticancer agents in human body. The anticancer nature is the coordination of metal ions with DNA molecules, i.e. the direct chelation of the metal ions with certain nucleophilic groups in DNA (such as oxygen sites from phosphates and nitrogen as well as oxygen sites from bases), causing the DNAs' damage in cancer cells, the DNAs were hindered during the processes of replication or transcription, the growing and division of the cancer cells were stopped, and resulted in their death.

When drug molecules (pre-anticancer molecules) enter into an organism, they will first undergo a series of processes including hydrolysis, transport and membrane-crossing, and then reach the nearby of target DNA molecule and form active intermediates which interact with DNA molecules directly and exert the anticancer activity. These active intermediates have a general cis-form of two-water-binding transitional state:

$$[cis\text{-}A_nM(H_2O)_2]^{m+},$$

A is stably binding hydrophobic group, n=1, 2 or more;

M is metal ion,

The number of water molecules binding to M must not be fewer than two and they must be lie in the ortho-position of the structure

The functions of the hydrophobic ligand A are:

(1) caring the whole molecule to cross membranes (including cell membranes and nucleus membranes), go through the bilipid bilayers;

(2) making the metal ion to move to the nearby of the base's cyclic-nitrogen sites and form covalent bonding.

Metal anticancer complexes are often electrophilic and may react with many cellular components, such as simple ions and molecules like $Cl^-$, $(HPO_4)^{2-}$, $OH^-$ and $H_2O$; amino acids, peptides and polyphosphates like His, Met, Cys, glutathione, metallothionein and ATP. Viewing from the coordination chemistry, metal complexes (including those of Pt) can bind to several types of possible biomolecules in the cell. But only the binding on DNA which lead to cell death is considered the most important. In the case of platnium complexes, it is quite clear that in the cells, after the relatively slow hydrolysis, cis-Pt have a preference for DNA over proteins and other molecules. Sadler and co-workers have shown that L-methionine increases the rate of reaction of 5'-GMP with cisplatin and that S-bound L-HMet in the adduct $[Pt(dien)(L\text{-}HMet\text{-}S)]^{2+}$ (dien=1,5-diamino-3-aazapentane) can be replaced by N7 of 5'GMP[31-33]. This work, together with the results of van Boom and Reedijk[34], who reported the intramolecular displacement of a Pt-bound thioether by a guanine nucleobase, suggest that novel routes to DNA platination from anticancer drugs may exit. Therefore it is conceivable that a methionine-containing protein or peptide could transport and transfer some platinum to DNA[33].

Thus, in a very simple model, the action process of a metal anticancer agent in an organism may be briefly summarized into following equations :

$$DX_2 + 2H_2O ==== [D(H_2O)_2]^{2+} + 2X^- \qquad (1)$$

$$[D(H_2O)_2]^{2+} + DNA ==== [DNA\text{-}D]^{2+} + 2H_2O \qquad (2)$$

$DX_2$ is the pre-anticancer molecule,
$[D(H_2O)_2]^{2+}$ is its active intermediate produced by hydrolysis,
DNA-D is DNA-drug complex.

DNA molecule is a two-pole molecule. Its surface is a negatively-charged backbone of phosphatepentose chains, in the inside of double helix there exist hydrophobic bases stacking layer by layer. For exerting its potency, the drug molecules must bind with the phosphate groups of DNA at first, and then, with the help of DNA' conformational dynamic changes (say, partial unwinding of the double helix), the lipophilic groups of the drug molecule may be drawn by DNA's hydrophobic sections, the nitrogen sites on DNA molecules may be exposed, the metal atom could invade into the internal part of DNA and coordinates with the bases. Oxygen site on the phosphate group has a higher negative charge relatively, it is a good donor with high electronegativity; so its action with the metal atom was caused mainly by static electricity, forming electrovalency, belongs to charge-controlling reaction.

Nitrogen atoms on the bases are donors with lower electronegativity, they react with the metal atom to form covalent bonds, belonging to orbital-controlling reactions.

It appears that these two interactions were brought forth one after another and coexisted. This interaction mode is in conformity with the principle of multiple binding by flexible fitting and maximum bonding, such a principle is generally accepted when the biochemical reactions are considered.

Through the study on hydrolysis mechanism and relationship between structure and activity of metal anticancer agents, we think that the following three points are key if the metal anticancer agents have activities:

(1) Appropriate hydrolysis rates of a complex;

(2) Forming the active intermediate $[cis\text{-}A_nM(H_2O)_2]^{m+}$,

(3) Forming of the coordination both with the oxygen of phosphate groups and with nitrogen of bases.

The molecules with high anticancer activities should not only produce active intermediates by the proper hydrolysis rates, but also bind to both oxygen of phosphate groups and nitrogen of bases in DNA, thus showing anticancer activity.

### 3.3 *Two-Pole Complementary Principle (TPCP)*

The similarities in chemical features of high active metal complexes infer that there must be some structural similarities in their molecular level. After comparison the structure-activity relationships of more than 200 metal anticancer complexes, including platinum complexes, we find they actually have some common structural features and acting laws for the molecules of metal anticancer agents. We generalize these factors into a rule named Two-Pole Complementary Principle(TPCP), which includes three aspects:

**(1) Two-pole complement in molecular structures:**

The drug molecules with anticancer activity always have two poles of hydrophilicity and hydrophobicity, positive and negative charges in their structures. Correspondingly, they will present easily-leaving groups and stable keeping groups in a solution. Such two-pole structures can lead the drug molecules not only to be dissolved in water and transported to the surface of the cell membranes, but also to cross the membranes by going through the lipid bilayers and arrive at the nearby of the target molecules.

**(2) Two-pole complement in the receptor-substrate action mode:**

The interaction between the drug molecule and its target molecule is always executed by forming an active intermediate which binds with oxygen sites (electrovalently) on phosphate groups and nitrogen sites (covalently) on purines, pyrimidines of DNA backbone through charge-controlling and orbital-controlling. That is a two-pole complement of electrovalent and covalent action modes.

**(3) Two-pole complement in the symmetry of the receptor-substrate system:**

The interaction characteristics of a chiral drug molecule with DNA behave as using the left hand enantiomer of the drug molecule binding with the right hand DNA, forming a two-pole complementary complex chirally.

From the above principles we can extend criteria which are used for assessment of activity of metal anticancer agents as follows:

(1) For the active intermediate of an anticancer agent molecule, its dipole moment does not equal to zero, i.e. $\mu_{ai} \neq 0$.

(2)The drug molecule should possesses both hydrophilic and hyrdophobic groups simultaneously. In an homologue of drugs, proper oil/water partition coefficients are needed and the hydrophobic group should not be too bulky .

(3) In the physiological liquid the drug molecule should have appropriate hydrolysis rate constants and at the near of DNA an active intermediate should combine at least two water molecules in cis-configuration.

(4) The metal ion of the interactive center must have appropriate Pearson hard-soft degree (i.e. proper valence and radium) as well as abundant valence shell orbital so that it may have an affinity not only for DNA's oxygen site on phosphate to form electrovalent bond but also for nitrogen sites on purine, pyrimidine to form covalent bond.

(5) The chiral drug molecule should be left hand enantiomer so that it can form a complementary structure with DNA molecule which is right hand chiral.

The above TPCP has generalized the molecular structure, action modes and steric selectivity for metal anticancer agents. This principle has some guidance for the design and synthesis of new metal antitumor drugs. However, for numerous various metal antitumor complexes, the action mechanisms may differ dramatically, and there are always some complex can't be accorded with the TPC Principle. And the TPC Principle itself may still has some incomplete aspects, and need to be further studied to make it more complete.

**Acknowledgment.**

The authors gratefully acknowledge Professor H.Taube and Dr. Zaiwei Li(Stanford University, CA) for their generous gift of the probe. The financial support from National Nature Science Foundation of China, National Nature Science Foundation of Shanxi Province, the Youth Science Foundation of Shanxi Province and the financial support from Shanxi Education Commission are greatly acknowledged.

**Reference**

1. J. Reedijk, *Handbook of Metal-Ligand Interaction in Biological Fluids,Bioinorganic Chemistry,* Vol. 2(G. Berthon Ed.) Marcel Dekker, Inc. p.967-989.(1995)
2. A. K. Saxena, F. Huber, *Coord. Chem. Rev.* **95,** 109(1989)
3. P. Koepf-Maier, H. Kopef, *Chem. Rev.* **87**, 1137(1987)
4. G. Sava, S. Zorzet, T.Giraldi, G. Eur. Zassinovich, *J. Cancer. Clin. Qnciol.* **20**, 841 (1984)
5. P. Koepf-Maier and D. Krahl, *Naturwiss* **68**, 273 (1981)
6. M. J. Clarke, *Prog. Clin. Biochem. Med.* **10**, 25 (1989)
7. A. S. Gonza, J. S. Casas, J. Sordo, *J. Inorg. Biochem.* **39**, 227(1990)
8. J. Reedijk, *Pure & Appl. Chem.* **59,** 181(1987)
9. K. J. Barnham, S. J. Berners-Price, Z. Guo, P d. S. Murdoch, P. J. Sadler, *Platinum and Other Metal Coordination Compounds in Cancer Chemothererapy* 2, ( H. M. Pinedo and J. H. Schornagel Ed.), Plenum Press, New York, pp 1-16 (1996)
10. W. I. Sundquist and S. J. Lippard, *Coord. Chem. Rev.* **100**, 293(1990)
11. G.Wilkinson, J. M. Birmingham, *J. Am. Chem. Soc.* **76**, 4281 (1954)
12. I.P.Evans, A.Spencer, and G.Wilkenson, *J. Chem. Soc., Dalton Trans.* **7**,204 (1973)

13. P. Newmann, *Annaler,* **653**, 157 (1962)
14. A. J. Crowe, P. J. Smith, *J. Organomet. Chem.* **224**, 223 (1982)
15. Z. W. Li, H. Taube, *Science* **256**, 210 (1992)
16. V.I.Ivanov, et al., *Biopolymers,* **12,** 89(1973)
17. M.J.Clarke,,et al., *Inorg. Chim. Acta* **124**, 13 (1986)
18. Zi-xian Lu et al., *Application of Circular Dicroism and Optical Rotatory Dispersion in Molecular Biology,* Science Pree, Beijing(1987)
19. J.B. Lepeoq, C. Paoletti, *J. Mol. Biol.* **27**, 87(1967)
20. M. Howe-Grant, K.C. Wu, W.R. Bauer, and S.J. Lippard, *Biochemistry* **15**, 4339(1976)
21. M.T. Carte and A.J. Bard, *J. Am. Chem. Soc.* **109**, 7528(1987)
22. J. Sun and D.K. Solaiman, *J. Inorg. Biochem.* **4,** 271 ( 1990)
23. P. Umapathy, *Coord. Chem. Rev.* **95**, 129(1989)
24. L.Y. Kuo, M.G. Kanatzidis, M. Sabat, A.L.Tipton and T.J.Marks, *J. Am. Chem. Soc.* **113,** 9027(1991)
25. R.N. Bose, R.D. Cornelius and R.E. Viola, *J. Am. Chem. Soc.* **108,** 403(1986)
26. T.G. Applaton, J.R. Hall, D.W. Neale and S.F. Ralph, *Inorg. Chem.* **25,** 720(1986)
27. M.D. Reily and L.G. Marzilli, *J. Am. Chem. Soc.* **108**, 8299 (1986)
28. M.D. Reily, T.W. Hambley and L.G. Marzilli, *J. Am. Chem. Soc.* **110**, 2999(1988)
29. L.Y. Kuo, M.G. Kanatzidis and T.J. Marks, *J. Am. Chem. Soc.* **109**, 7207( 1987)
30. E. Alessio, Y.Xu, S. Cauci, G. Mestroni, F. Quadrifoglio, P.Viglino, L.G. Marzilli, *J. Am. Chem. Soc.* **111,** 7068 (1989)
31. K. J. Barnham, M. I. Djuran, P. d. S. Murdoch, P. J. Sadler, *J. Chem. Soc. Chem Commun.* 721(1994)
32. K. J. Barnham, M. I. Djuran, P. d. S. Murdoch, J. D. Ranford, P. J. Sadler, *J. Chem. Soc. Dalton Trans.* 3721(1995)
33. K. J. Barnham, M. I. Djuran, P. d. S. Murdoch, J. D. Ranford, P. J. Sadler, *Inorg. Chem.* **35**, 1065(1996)
34. S. S. G. E. van Boom and J. Reedijk, *J. Chem. Soc. Chem. Commun.* 1397(1993)

**Received: August 8, 1997 - Accepted: August 25, 1997 -**
**Received in revised camera-ready format: January 19, 1998**

